# G4 Oligonucleotide-Based Chaperones of Heterogeneous Nuclear Ribonucleoprotein A1

**DOI:** 10.3390/ijms262010104

**Published:** 2025-10-17

**Authors:** Elizaveta Malakhova, Julia Svetlova, Iuliia Pavlova, Sabina Alieva, Vyacheslav Severov, Nikolay Barinov, Dmitry Klinov, Tatiana Vedekhina, Anna Varizhuk

**Affiliations:** Lopukhin Federal Research and Clinical Center of Physical-Chemical Medicine of Federal Medical Biological Agency, Malaya Pirogovskaya, 1a, 119435 Moscow, Russiawws83@yandex.ru (V.S.); neglect1@yandex.ru (T.V.)

**Keywords:** chaperone, G-quadruplex, ribonucleoprotein, aggregation, proteinopathy

## Abstract

Pharmacological chaperones of heterogeneous nuclear ribonucleoproteins (hnRNPs) show promise as potential neuroprotective drug candidates. They are expected to prevent the accumulation of neurotoxic hnRNP biocondensates and aggregates, which are hallmarks of severe degenerative diseases. Here, we present the first rational design of oligonucleotide chaperones of hnRNP A1. This design was inspired by previous studies on the specificity of the RNA recognition motif (RRM) and the RGG motif of hnRNP A1 for endogenous nucleic acids. To obtain robust and specific chaperones, we combined an RRM-binding sequence with an RGG-binding G-quadruplex oligonucleotide that inhibits hnRNP A1 aggregation and introduced various modifications into the sugar-phosphate backbone of the oligonucleotide. Modifications that locked the RRM-binding sequence in a conformational state characteristic of RNA improved chaperone affinity and activity. The former was assessed using microscale thermophoresis assays, while the latter was evaluated using fluorimetry and microscopy. The leading chaperone bound to hnRNP A1 at micromolar concentrations and inhibited the assembly of its condensates and amyloid-like aggregates (fibrils) by over 90%.

## 1. Introduction

Endogenous proteins that stabilize, maintain or restore the tertiary and quaternary structures of other proteins are commonly known as chaperones [1]. Examples include heat shock proteins and transportines. Recently, the term “chaperone” has also been applied to oligonucleotides and small molecules that perform similar functions in the context of chaperone therapy for pathologies driven by protein misfolding [2,3]. These pathologies include neurodegenerative diseases classified as proteinopathies and metabolic disorders, most of which are enzymopathies (e.g., lysosomal storage diseases) [4]. Accumulation of misfolded proteins causes endoplasmic reticulum (ER) stress, damages cellular membranes and impairs intracellular signaling and transport [5]. The latter is critical in neurons due to their unique morphology [6].

Proteinopathies are typically driven by mutations or aberrant post-translational modifications that promote protein misfolding [7]. A lack of endogenous hydrotropes [8] or chaperones [9] is an equally significant risk factor and has been considered when developing related therapeutic strategies [10]. One of the first strategies tested in model systems involved activating endogenous chaperone expression [11], which, is some cases, promoted oncogenic transformation [12]. Other strategies require the use of exogenous chaperones, which can be classified as either chemical (non-selective solubilizers that prevent global EPR stress) or pharmacological (selective chaperones that recognize specific misfolded proteins) [13]. The latter restore or maintain protein folding through allosteric action or by shielding oligomerization sites. Notable examples of pharmacological chaperones include small molecules that target the prion proteins [14] and proteins involved in the pathogenesis of Parkinson’s disease [15,16,17] or Alzheimer’s disease [18,19].

Key targets of oligonucleotide chaperones are heterogeneous nuclear ribonucleoproteins (hnRNPs), which regulate RNA processing and transcription. Each member of this family contains at least one RNA-recognition motif (RRM) and a conformationally disordered low-complexity region (LCR) rich in glycine, proline, acidic amino acid residues, or RGG repeats [20]. Polar but uncharged LCRs are designated as prion-like domains (PLDs) [21]. These domains are responsible for hnRNP phase transitions [22,23], which include the formation of biocondensates through liquid–liquid phase separation (LLPS) [24,25], the gelation or solidification into amorphous aggregates, and the self-assembly into amyloid-like cross-beta fibrils (amyloidization) [26].

A well-known example of a native oligonucleotide chaperone is the promoter-associated GU-rich RNA. First, this RNA was shown to prevent the aggregation of hnRNP TDP-43, supposedly in a manner reminiscent of chemical chaperones [27]. Second, the GU-rich RNA was found to inhibit the aggregation of hnRNP FUS under mechanical stress [28]. These findings could not be explained by the sequence-specific recognition of the RNA by RRMs alone, suggesting the presence of alternative RNA-binding sites in the hnRNPs. Additionally, structure-specific rather than sequence-specific binding could not be ruled out because GU-rich sequences may undergo noncanonical folding. The likely alternative RNA-binding sites of hnRNPs are RGG motifs [29,30,31]. Importantly, these intrinsically disordered motifs can interact with proximal PLDs. It has been hypothesized that RNA molecules compete with PLDs for RGG, thereby disrupting the intra- and intermolecular contacts required for hnRNP aggregation [32].

The presumed link between oligonucleotide secondary structures and chaperone activity has been verified and partially confirmed in recent studies on an expanded set of hnRNPs [33]. Specifically, G-rich DNA and RNA sequences that adopt G-quadruplex (G4) topologies [34,35,36] showed a holdase-like activity toward RGG- and PLD-containing hnRNPs FUS, TDP-43, and hnRNP A1 [37,38], i.e., inhibited transitions of these proteins into condensates, amorphous aggregates, or cross-beta fibrils [39,40]. Such condensates, aggregates, and fibrils are recognized as markers of amyotrophic lateral sclerosis (ALS) [41,42] and related neurodegenerative diseases [43,44]. Therefore, G4 chaperones of FUS, TDP-43, and hnRNP A1 could be considered as potential neuroprotectors (e.g., anti-ALS drug candidates). However, they may exhibit a wide range of side effects due to the diversity of the G4 interactome. Here, we addressed this issue when designing G4-based chaperones of hnRNP A1 by complementing the RGG-binding G4 sequence with an RRM-binding sequence. In the future, the same principle can be adapted for FUS and TDP-43.

## 2. Results

### 2.1. Chaperone Design

**Figure 1:**
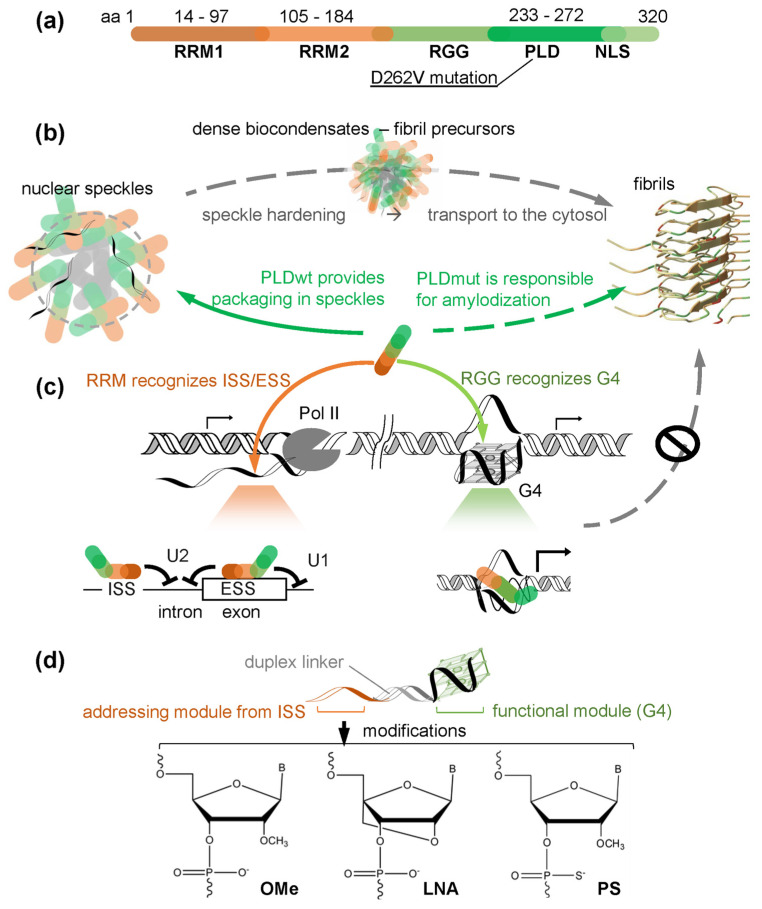
Functions of hnRNP A1 and the chaperone design strategy. (**a**) Domain structure of hnRNP A1. RRM—RNA-binding motif, RGG—arginine/glycine-rich motif, PLD—prion-like domain (the site of the amyloidization-promoting mutation is marked), NLS—non-canonical nuclear localization signal. (**b**) Phase transitions of hnRNP A1 under normal (solid arrow) and pathological (dashed) conditions. (**c**) The relevance of hnRNP A1 motifs for the regulating transcription and splicing. The red arrow indicates RRM binding to intronic/exon silencing sites (ISS/ESS) of pre-mRNA, which results in the repressed spliceosome assembly at nearby splice sites. The green arrow indicates RGG binding to promoter G4s, which results in G4 unwinding and may facilitate transcription. The G4s/ISS-bound hnRNP is monomeric. (**d**) Chaperones of hnRNP A1: a schematic representation of the two-step design strategy. The two steps include combining the ISS -binding sequence (the addressing module) with a promoter G4 (the functional module) and introducing backbone modifications. The bottom panel shows the three types of modifications tested in this study: 2′-O-methyl oligonucleotide derivatives (OMe), locked nucleic acids (LNA), and phosphorothioate nucleic acid mimics (PS).

The rationale behind the design of the oligonucleotide chaperones was inspired by the domain structure of hnRNP A1 (Figure 1a), the role of its RGG-PLD fragment in phase transitions (Figure 1b), and the specificity of the RGG motif and the adjacent RRM to DNA and RNA (Figure 1c).

The chaperones were designed to contain two key units: an RGG-binding functional module (G4) and a single-stranded RRM-binding addressing module. These modules were connected by a duplex linker (Figure 1d).

The functional module was chosen based on the studies of hnRNP A1 interactions with genomic G4s enriched in promoters [45] and microsatellites [46]. HnRNP A1 binds to genomic G4s when recruited from nuclear speckles to euchromatin (Figure 1b) [47]. There, it assists in transcription activation by destabilizing G4 structures, presumably through the helicase-like activity of its RGG motif (Figure 1c) [48,49]. Promoter-bound hnRNP A1 is protected from aggregation, likely because G4-RGG contacts prevent RGG-PLD contacts [37]. We assumed that a compact promoter G4 would be a robust chaperone scaffold. Thus, we selected a minimal G4 motif enriched in promoters, d(G_3_TG_3_TG_3_TG_3_) [50], as a candidate functional module. A well-characterized hnRNP-binding G4 motif from the *KRAS* promoter, d(AG_3_CG_2_TGTG_3_AAGAG_3_AAGAG_5_AG_2_) [48], was used as a control functional module. These modules are expected to exhibit holdase-like activity in both deoxyribo and ribo variants [37,38].

The addressing module was chosen based on the studies of hnRNP A1 interactions with intronic/exonic regulatory elements, which occur during the co-transcriptional processing of pre-mRNA and determine the outcome of alternative splicing (Figure 1c) [51]. Among these interactions, the sequence-specific recognition of intronic splicing silencers (ISS) by hnRNP RRMs is arguably the most important [45]. We reasoned that combining the G4 motif with an ISS motif would increase chaperone affinity and selectivity towards hnRNP A1. Thus, we selected an ISS motif UCAGUU, whose interaction with RRM2 was confirmed by NMR spectroscopy data [52], as the addressing module. Structural data suggest that the key ISS-RRM2 contacts involve CAG bases. Thus, the deoxyribo variant of the addressing module can partially preserve the affinity for RRM2 characteristic of the ribo variant.

The duplex linker between the two chaperone modules was taken from an artificial hairpin structure sc1-RNA, which is an aptamer for the RGG-containing protein FMRP [53]. The loop of this hairpin folds into a G4 structure, and this folding is stabilized by the adjacent duplex (i.e., the loop stem GCUGC/GCAGC). According to the X-ray diffraction data [53], the duplex also forms contacts with the RGG beta-turn in the sci-RNA-FMRP complex. We expected this duplex to have a similar G4-stabilizing effect within a double-module chaperone. Additionally, we expected it to form contacts with RGG in a chaperon-hnRNP A1 complex. One limitation of our study is the possibility of suboptimal duplex length. In future, this may need to be fine-tuned with respect to the RRM-RGG junction of hnRNP A1 or other target proteins. The RRM2-RGG junction of hnRNP A1 (aa 185-217) is Gly-rich and disordered, so we expected it to be partially looped out in the hnRNP-chaperon complex, similar to the looping of the inter-RRM junction in the complex with telomeric DNA [54] (PDB ID: 2UP1) and other native sequences [52]. This suggests sufficiency of the short duplex linker in the double-module chaperon but requires verification by NMR- or X-ray diffraction studies.

The resulting chaperones include double-module ribo (chap-RNA) and deoxyribo (chap-DNA) oligonucleotides with the minimal G4, a positive control with the *KRAS* G4 (cntr-KRAS) and the same addressing module, and a semi-negative control lacking the addressing module (cntr-noISS). All sequences are presented in Table 1. We expected chap-RNA to outperform chap-DNA in terms of affinity [45,53]. However, susceptibility to nuclease hydrolysis complicates the testing and potential application of ribooligonucleotides. Chap-DNA, especially its functional module, must be more stable. The compact G4 is typically rather resistant to nucleases [55,56], whereas the addressing module and linker duplex are prone to rapid degradation in biological environments.

In this regard, we designed additional chaperon candidates based on chap-DNA and chap-RNA (Figure 1d) by introducing backbone modifications in the addressing module and the linker (chap-PS/OMe/LNA) or along the entire chain (chap-tPS/tOMe/tLNA). We chose the phosphorothioate modification of the inter-nucleotide bonds (PS) to protect the chaperones from nuclease hydrolysis. We also substituted deoxyribose residues for “conformationally restricted” analogs with 2′-O-4′-methylene bridges (LNA) or 2′-O-methyl groups (OMe). These modifications enhance nuclease resistance, ensure optimal geometry of the addressing module by fixing the sugar in the 3′-endo conformation, and stabilize the linker duplex [57,58,59]. All chaperones (Table 1) were tested for target binding affinity, secondary structure conservation and chaperone activity.

### 2.2. Analysis of Chaperon Secondary Structures and Affinity to hnRNP A1

**Figure 2:**
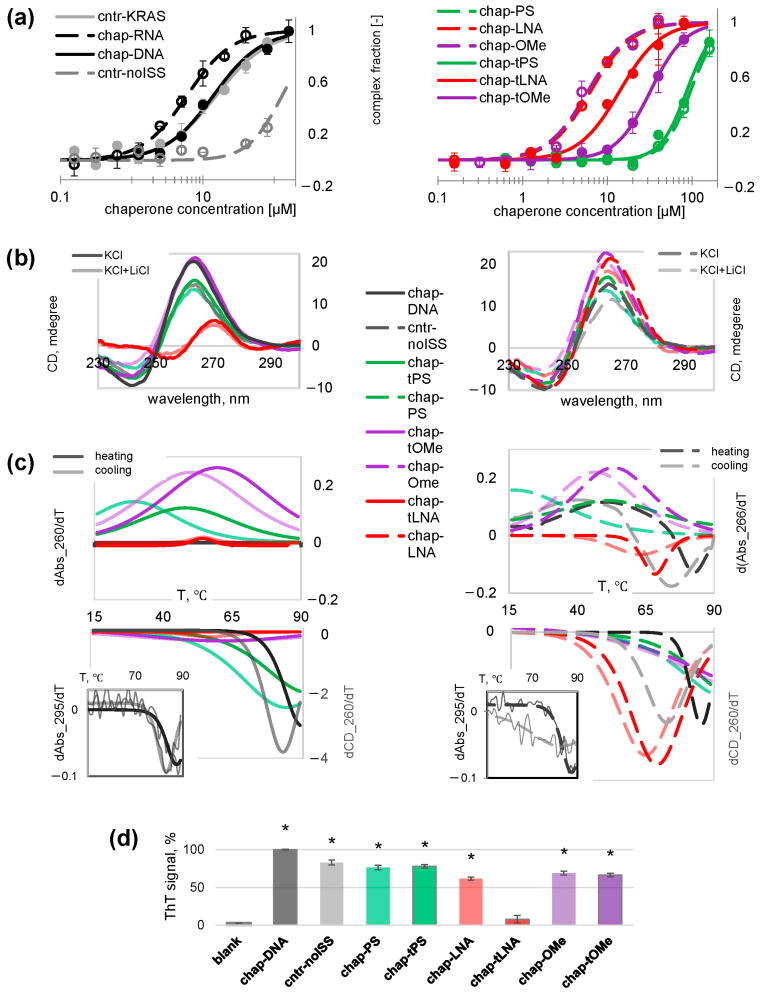
Chaperone affinity assays and secondary structure verification. (**a**) Analysis of affinity to hnRNP A1 (MST assays): binding curves of the native chaperon and the control oligonucleotides (left) or fully/partially modified chaperones (right) in the working buffer containing 150 mM KCl. (**b**) Secondary structure verification: CD spectra of the native and partially modified chaperones (left) or fully modified chaperones and the control oligonucleotide (right) in the working buffer containing 10 mM KCl (dark lines) or 10 mM KCl and 140 mM LiCl (pale lines). (**c**) Verification of the thermal stability of the chaperone secondary structures: first derivatives of denaturation (dark lines) and renaturation (pale lines) profiles obtained in the working buffer containing 10 mM KCl and 140 mM LiCl. Panels (**b**,**c**) have a joint legend. (**d**) Verification of G4 folding (ThT assays). The ThT signal is the fluorescence intensity of ThT in the chaperone solution. Conditions: 10 μM oligonucleotide and 20 μM ThT, 37 °C. * *p* < 0.001 (significant difference from blank, Dunnett’s test).

The affinity of the chaperones and the control oligonucleotides for hnRNP A1 was evaluated using microscale thermophoresis (MST) assays in a pseudophysiological buffer solution. Figure 2a and Table 1 show the binding curves and dissociation constants (Kd) of the complexes, respectively. The control single-module oligodeoxyribonucleotide (cntr-no ISS) exhibited minimal affinity for hnRNP A1. Double-module oligodeoxyribonucleotides chap-DNA and cntr-KRAS exhibited similar moderately high affinities (Kd values of approximately 15 μM) and were inferior to chap-RNA (Kd = 6 ± 1 μM). These results confirm the benefits of the addressing module and the advantages of native RNA chaperones over DNA ones.

Among the modified chaperones, chap-LNA and chap-OMe exhibited the highest affinity, similar to that of chap-RNA. The inferior performance of the fully modified chap-tLNA and chap-tOMe analogs compared to the partially modified ones suggests possible distortions of the secondary structure of the functional module that prevent its efficient contacts with RGG. Both PS-modified chaperones (chap-PS and chap-tPS) were inferior to chap-DNA, which also suggests altered secondary structures.

Secondary structures of the chaperons and the control oligodeoxyribonucleotides were investigated by circular dichroism (CD) spectroscopy in buffer solutions containing 10 mM KCl, with or without the addition of 140 mM LiCl (Figure 2b). Potassium, but not lithium, ions promote G4 folding [60]; for duplexes, however, the type of cations is not crucial. The excess of lithium ions was expected to yield moderately stable rather than extra-stable G4s and facilitate their comparative analysis.

The CD spectra of all the oligonucleotides, except chap-tLNA, were consistent with the secondary structure outlined in Figure 1d. They contained a positive band around 265 nm and a small negative band around 245 nm, which are characteristic of parallel-stranded G4s [61]. The amplitudes of these bands decreased slightly to moderately in the presence of LiCl, which is consistent with the presence of G4s. The absence of the G4 signature in the chap-tLNA spectrum does not contradict previous studies [62] and explains the reduced affinity of this modified oligonucleotide to the target protein (Figure 2a).

The dominance of the G4 signature hindered the accurate analysis of linker folding. In the spectra of chap-DNA, chap-PS, and chap-tPS, the positive band at 280 nm expected for the B-form linker duplex [61] was barely visible as a shoulder of the G4-peak. In the spectra of chap-RNA, chap-OMe and chap-LNA, the signature of the expected A-form duplex [61] overlapped with the G4 signature. However, thermal denaturation/renaturation experiments in the presence of LiCl allowed us to distinguish the duplex and G4 structures in most cases (Figure 2c).

Generally, duplex melting is accompanied by a significant hyperchromism at 260 nm [63], while CD changes at 260 nm are minimal [61]. G4 melting is typically accompanied by hypochromism at 295 nm and, in some cases, at 265 nm [63,64], as well as a decrease in CD at 260 nm [65]. Thus, the chaperone denaturation/renaturation profiles obtained through absorbance monitoring at 260 nm had positive first derivatives and mainly indicated changes in the duplex folding/unwinding (Figure 2c, upper panel). In contrast, the profiles obtained through CD monitoring at 260 nm had negative first derivatives and indicated G4 folding/unwinding (Figure 2c, lower panel).

G4 folding of native oligonucleotides was additionally confirmed by monitoring absorbance changes at 295 nm. The signal-to-noise ratio was insufficient for proper analysis of the modified chaperones. For chap-DNA and cntr-noISS, however, both the denaturation and renaturation profiles (Figure 2c, side plots) agreed reasonably well with the respective CD-based profiles (Figure 2c, main bottom plots). The difference between denaturation and renaturation (hysteresis) was moderate (<10 °C) for chap-DNA and most of the modified chaperons. Significant (>15 °C) hysteresis observed for cntr-noISS, chap-PS, and chap-tPS suggests the possibility of intermolecular folding [65], which explains the reduced affinity of these oligonucleotides for the target protein (Figure 2a).

The melting temperatures (T_1/2_) of the G4s and duplexes were calculated as the mean values of the inflection points of the respective denaturation and renaturation profiles. The T_1/2_duplex_ values of LNA-modified oligonucleotides could not be determined, while those of other chaperons ranged from 30 °C to 55 °C. The T_1/2_G4_ values of the native oligonucleotides were 79 ± 2 °C (cntr-noISS) and 83 ± 2 °C (chap-DNA). Among the modified chaperons, only chap-LNA exhibited unambiguous G4 melting (T_1/2_ = 66 ± 2 °C). The G4s of other modified chaperones were either completely unfolded at room temperature (chap-tLNA) or mostly folded even at 90 °C (chap-OMe, chap-tOMe, chap-PS, chap-tPS).

The incomplete denaturation of the modified chaperones in the presence of LiCl could be due to the extremely high stability of the selected G4 scaffold [66], so further verification by an independent method was necessary. Thus, we verified G4 folding using fluorimetry in the presence of a low molecular weight dye thioflavin T (ThT) [67]. This dye does not fluoresce in the free state. In peptide- and protein-free oligonucleotide solutions, it binds to G4s with high specificity. The formation of the complex is accompanied by ThT fluorescence enhancement, or “light-up”. At micromolar ThT concentrations, the light-up effect is proportional to the G4 fraction. As evident from Figure 2d, the ThT test confirmed the presence of folded G4s in all chaperones, except for chap-tLNA, whose light-up effect was only slightly higher than that of the scrambled sequence (scr-LNA) used previously as a control non-structured LNA [68,69].

The above analysis of the structure-affinity relationship can be summarized as follows:

- Oligonucleotides chap-LNA and chap-OMe, which contain RNA-mimicking backbone modifications in the addressing module, retain G4 folding in the functional module and exhibit the same affinity for the target protein as chap-RNA.
- Oligonucleotides chap-tLNA and chap-tOMe, which contain RNA-mimicking backbone modifications throughout the chain, have reduced affinity for the target protein, likely due to loss of the G4 structure and changes in its geometry, respectively.
- Oligonucleotides chap-PS, chap-tPS, cntr-noISS have low affinity for the target protein, likely due to intramolecular folding.

### 2.3. Analysis of Chaperone Activity

Verification of chaperone activity included assessing its effects on hnRNP A1 cross-beta fibrils (amyloid-type aggregates) and condensates (aggregate precursors). First, we focused on the condensates. In cells, hnRNP A1 and other hnRNPs that are temporarily unengaged in transcription or splicing co-separate with SR-rich proteins, non-coding RNA, pre-mRNA, poly-A RNA, and small nuclear RNA into nuclear condensates known as splicing speckles [70]. These speckles are loosely organized throughout interphase. Their aberrant gelation or hardening under pathological conditions precedes hnRNP redistribution into the cytoplasm, where the dense condensates can mature into solid aggregates (Figure 1b). To evaluate the influence of chaperones on this process, we employed the previously described speckle mimics (model condensates) [38].

**Figure 3:**
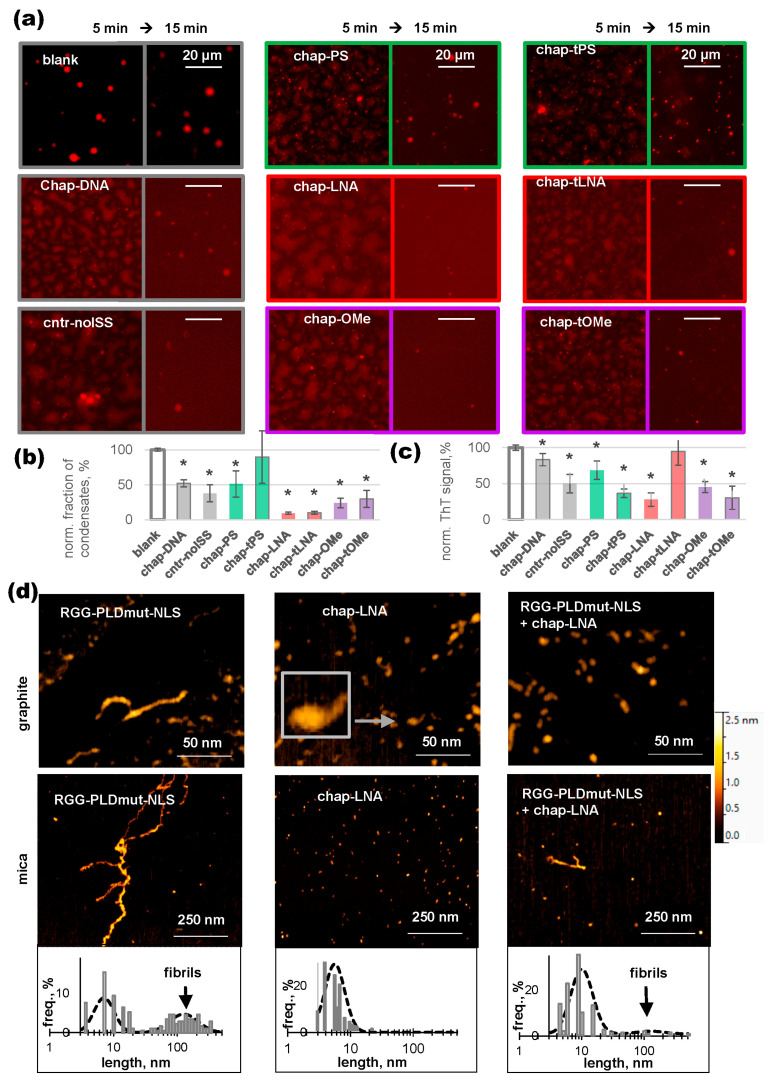
Chaperone activity assays. (**a**) Chaperone-induced dissolution of hnRNP A1 condensates. Conditions: 6 μM hnRNP A1 (5% labeled), 1 mg/mL SRSFfr, 3 mg/mL RNA, and 6 μM chaperone in working buffer containing 10% PEG. (**b**) Summary of the effects of the chaperones on the condensates. * *p* < 0.001 (significant difference from blank, Dunnett’s test). (**c**) Summary of the effects of the chaperones on the fibrils (ThT test). Conditions: 10 μM pRGG-PLD-NLS peptide, 10 μM chaperone, and 20 μM ThT in the working buffer. Norm. ThT signal = (ThT FI_mixture—ThT FI_chaperone)/ThT FI_peptide. (**d**) Examples of AFM scans of the peptide, chap-LNA, and their mixture on modified graphite (top) or mica (middle) and object size distribution plots for mica scans (bottom).

The model condensates were assembled in vitro from key speckle components and their analogs: recombinant hnRNP A1 labeled with a red fluorescent dye, model RNA of mixed composition, and a fragment of SR-rich splicing factor 1 (SRSF^fr^). The mixture of these components in a pseudophysiological buffer undergoes spontaneous LLPS in the presence or absence of the crowding agent polyethylene glycol (PEG), yielding dense or loose condensates, respectively [38]. In this study, the condensates were assembled in the presence of PEG and visualized as bright, spherical droplets enriched with RED-hnRNP A1 by fluorescence microscopy in the absence of chaperones (Figure 3a, “blank”).

The addition of chap-DNA partially dissolved the condensates, similar to what was previously reported for G4-RNA [38]. This dissolution occurred through an intermediate spinodal phase (see 5 min images in Figure 3a). The effect of the control oligonucleotide, cntr-noISS, was slightly less pronounced: more dense condensates remained in the spinodal phase. This is consistent with the decreased affinity of cntr-noISS for hnRNP A1. To quantitatively analyze the inhibitory effects, we calculated the volume fraction of the remaining condensates after 15 min of incubation based on the total area of condensate projections on the glass support [71]. The results are summarized in Figure 3b. The decrease in the condensate fraction (50–60%) was significant for both of the native chaperones (*p* < 0.001).

The fully modified chaperone chap-tPS had an insignificant effect on the condensate fraction. Its partially modified analog, chap-PS, demonstrated inhibitory activity similar to that of chap-DNA. In contrast, chap-LNA, chap-tLNA, chap-OMe, and chap-tOMe outperformed chap-DNA (Figure 3a,b). Notably, the effect of chap-tLNA was apparent even though its G4 module was unfolded (Figure 2). Further investigation is required to address this issue. We hypothesize that, provided the addressing module is recognized by the RRM, even the unfolded functional module may form weak electrostatic contacts with RGG, competing with longer model RNA and thus inhibiting the formation of dense condensates. Another possibility is that the enhanced rigidity of the LNA backbone reduces the probability of LLPS [72]. Overall, the LLPS-inhibiting activity of the chaperones reasonably correlated with their binding affinity. The most pronounced effect (90 ± 10% inhibition) was observed for chap-LNA, which is one of the top hnRNP A1 binders (Figure 2a).

Next, we analyzed the effects of the chaperones on amyloid-like fibrils. Amyloidization can occur at the interface between condensates and the bulk solution [39,40], but this process is slow, taking days. RRM-free partial hnRNP A1 forms fibrils within hours and this process can be accelerated by cross-beta-stabilizing mutations in PLD, such as the ALS-promoting D262V mutation (Figure 1a) [44]. We modeled the amyloidization of hnRNP A1 using a fragment of the protein that constitutes the core of the cross-beta structure (amino acids 218–272). This fragment comprises the RGG motif, the mutant PLD, and the non-canonical nuclear localization signal (NLS). The amyloidization of the RGG-PLDmut-NLS sequence has been verified previously [38]. In the absence of chaperones, LLPS-promoting SR-rich proteins, or model RNA, RGG-PLDmut-NLS formed fibrils within 30 min of incubation at 37 °C at a medium micromolar concentration, as confirmed by ThT light-up (Figure 3c).

Although ThT assays are the gold standard for amyloidization studies, the fact that ThT lights up with both fibrils and G4s complicates signal interpretation in the presence of the oligonucleotide chaperones [73,74]. To address this issue, we mixed ThT, each chaperone, and RGG-PLDmut-NLS at a ratio of 2:1:1 to allow for the simultaneous formation of ThT-G4 and ThT-fibril complexes. In the absence of chaperone activity, the sum of the G4- and fibril-induced light-up effects was expected. Thus, we subtracted the ThT signal of a free chaperone sample (Figure 2d) from the signal of its mixture with RGG-PLDmut-NLS. We then normalized the resulting value by the ThT signal of the free RGG-PLDmut-NLS sample. The results are shown in Figure 3c.

Decreased normalized values indicate non-additivity of the ThT light-up effects, likely due to chaperone activity. We emphasize that this assay is indirect and regard it as preliminary activity screening. Signs of chaperone activity were observed for all tested oligonucleotides except for chap-tLNA, which has an unfolded G4 module, and the unstructured control modified oligonucleotide scr-LNA (Figure 3c). The reduction in the normalized ThT signal was particularly pronounced in the case of chap-LNA (>70% apparent inhibition), so it was selected for further activity tests.

The effect of chap-LNA on the amyloidization of RGG-PLDmut-NLS was verified using atomic force microscopy (AFM). This method allows for the direct visualization of RGG-PLDmut-NLS fibrils, monomers, and complexes with chaperones. However, differences in their adsorption on an AFM support may affect visualization results, which is a potential limitation in terms of the quantitative analysis. To address this issue, we used different supports: modified graphite (top panel in Figure 3d) and mica (middle panel in Figure 3d). The results were consistent with each other and with the results of the ThT assay.

On both supports, chaperone-free RGG-PLDmut-NLS was visualized as a mixture of irregularly shaped small granules (monomers/oligomers) and rod-like structures (protofibrils and fibrils). The latter had lengths up to 500 nm and an apparent height of about 2.3 nm (Figure 3d, left panels), which is consistent with previous reports [38]. The typical morphology of chap-LNA (Figure 3d, top middle panel) was a 1–1.5 nm granule (the functional module) with a protruding “tail” (the addressing module), which agrees with the presumed chaperon structure (Figure 1d). The mixture of RGG-PLDmut-NLS and chap-LNA also contained granules attributable to individual components or complexes. However, the number of rod-like structures in this mixture was minimal (Figure 3d, right panels).

For a semi-quantitative analysis, we designated all rod-like structures of ≥20 nm in length as fibrils and calculated their fraction among all objects (Figure 3d, bottom panels). Chap-LNA decreased this value by over 90%, i.e., from approximately 40% to 1–2%. Thus, the AFM data are consistent with the ThT assay and confirm that chap-LNA inhibits amyloidization.

The overall results of the activity studies can be summarized as follows:

- Chap-LNA and chap-OMe, the chaperones with the highest affinity for hnRNP A1, efficiently inhibited condensate formation and amyloidization according to fluorescence microscopy and ThT assays.
- PS-modified chaperones inhibited amyloidization, according to ThT assays, but were inferior to other modified chaperones in terms of their effects on the condensates according to fluorescence microscopy assays.
- AFM assays confirmed the inhibitory effect of the leading chaperone, chap-LNA, on amyloidization.

## 3. Discussion

We designed oligonucleotide chaperones of hnRNP A1 based on a minimal sequence motif that forms a stable, three-tetrad G4 structure with single-nucleotide loops. We avoided lengthy loops because they allow for conformational polymorphism, which may reduce reproducibility. However, according to previous studies of hnRNP A1 complexes with the telomeric G4 and its analogs, loops might be involved in protein recognition. An analog with 1′,2′-dideoxyribose residues in the loops exhibited reduced affinity for the target protein, which highlights the role of loop nucleobases [75]. According to our data (Figure 2a, Table 1), single-nucleotide loops are sufficient. The native chaperone containing the minimal G4 (chap-DNA) showed the same affinity for hnRNP A1 as its analog with the G4 from the *KRAS* promoter (cntr-KRAS).

Previous studies of G4 holdase activities toward hnRNPs [37,38] and related aggregating proteins [76] revealed the importance of the G4 topology. Parallel-stranded intramolecular G4s are generally more active than antiparallel ones; however, in some cases, conformational dynamics and hybrid topologies are beneficial [35]. In this study, all of the tested chaperones, except for chap-LNA, contained parallel-stranded G4s (Figure 2b). Signs of intermolecular folding (profound hysteresis) were evident only for PS-modified chaperones (Figure 2c). These chaperones showed reduced affinity (Figure 2a) and/or activity (Figure 3c). Thus, although incomplete, our analysis of the structure-affinity/activity relationship agrees reasonably well with the literature.

When discussing holdase activity, it is helpful to distinguish the effects of chaperones on different types of phase transitions. The chaperones presented in this study suppressed both solidification and liquid–liquid phase separation (LLPS), though to varying extents. Interestingly, examples of G4s activating LLPS have been previously described [77,78,79]. For instance, the formation of condensates is enhanced in the presence of *C9Orf72* transcripts enriched with G4-forming G_4_C_2_ repeats [80]. Expansion of these repeats is a risk factor for developing ALS and related neurodegenerative diseases, alongside hnRNP mutations [81]. Notably, LLPS-promoting G4s are mostly multimeric or tandem, and their effects can be explained by increasing multivalency in native RNA/DNA. This does not contradict the holdase activity of isolated monomeric G4s.

The use of isolated G4s as chaperones is limited due to their low selectivity and biostability. The proposed approach to overcoming these limitations—namely, combining a functional G4 with a backbone-modified addressing module—is a step forward toward targeted chaperone therapy. The leading double-module chaperone of hnRNP A1 (chap-LNA) may require optimization, such as fine-tuning of the linker, and further investigation. Nevertheless, it is a promising scaffold for developing hnRNP A1-targeting anti-ALS drug candidates. The fact that chap-LNA outperformed native and single-module analogs in most in vitro tests underscores the potential of the proposed chaperone design approach.

## 4. Materials and Methods

### 4.1. Oligonucleotides

Oligodeoxyribonucleotides and their modified derivatives were obtained from Litech LLC (Moscow, Russia) and purified by high-performance liquid chromatography (HPLC) with UV detection at 260 nm. HPLC was performed using a 250 × 4.6 Hypersil C18 column (Thermo Fisher Scientific, Waltham, MA, USA) and a linear gradient of acetonitrile in a 0.1 M ammonium acetate buffer solution at a temperature of 50 °C and a flow rate of 0.85 mL/min. The dimethoxytrityl protecting group was removed by treating the sample with 80% acetic acid for 30 min. The detritylated oligonucleotides were subjected to additional HPLC purification using a reduced gradient of acetonitrile in the same ammonium acetate buffer and then precipitated with cold ethanol. The final purity of all oligonucleotides was at least 95% (HPLC). Time-of-flight mass spectrometry with matrix-assisted laser desorption/ionization on a Microflex spectrometer (Bruker, Billerica, MA, USA) additionally confirmed the structure and purity. The oligoribonucleotide was obtained from Genterra (Moscow, Russia). Mixed RNA from Torula yeast was obtained from HiMedia Laboratories (Thane, India).

### 4.2. Proteins and Peptides

The recombinant hnRNP A1 protein with a 6xHis tag was obtained from AMSBIO (Abingdon, UK). The His-tag labeling of this protein with a red dye (an AlexaFluor 647 analog) was performed using the RED-Tris-NTA kit according to the manufacturer’s protocol (NanoTemper Technologies, Munich, Germany). The partial mutant hnRNP A1 (aa 218-272), which comprises the RGG motif, PLD with the ALS-promoting mutation D262V [44], and NLS (RGG-PLD^mut^-NLS), as well as the auxiliary SR-rich peptide SRSF^(fr)^ for co-condensation with hnRNP A1 were obtained by solid-phase synthesis and purified by according to the previously described protocol [38]. Sequences of RGG-PLD^mut^-NLS and SRSF^(fr)^ were also taken from [38].

RGG-PLD^mut^-NLS: RGGNFSGRGGFGFGGSRGGGGGYGGSGDGYNGFGFGNDGSNDGSNFGGGGSYNVFGNYNNNNNQSSN);

SRSF^(fr)^: PRSPSYGRSRSRSRSRSRSRSRSNSRSRSYSP.

### 4.3. Absorption and Circular Dichroism (CD) Spectroscopy

Absorption and CD spectroscopy were used to verify the secondary structures of the oligonucleotides. The oligonucleotides were dissolved to a final concentration of 1 μM in a 40 mM HEPES-KOH buffer solution (pH 7.0) supplemented with 10 mM KCl, and optionally with 140 mM LiCl. The solutions were heated to 90 °C for 5 min and cooled on ice to facilitate intramolecular folding prior to all measurements. CD spectra were recorded in the 220–330 nm range at 20 °C using a Chirascan spectrophotometer (Applied Photophysics, Surrey, UK) in quartz cuvettes with an optical path length of 1 cm. The denaturation/renaturation profiles with absorbance/CD detection at 260/295 nm were recorded in continuous heating/cooling mode at a rate of 1 °C/min and approximated by a sigmoid function using ProData Viewer software (version 4.4) (Applied Photophysics, Surrey, UK). All experiments were performed in duplicate. The spectra and the profiles were averaged after baseline subtraction.

### 4.4. Fluorimetry

Fluorimetry with thioflavin T (ThT), a fluorogenic probe for amyloid fibrils [71] and G4s [73] was used to track the amyloidization of the hnRNP A1 fragment RGG-PLDmut-NLS and to confirm the G4 folding of the oligonucleotide chaperones. For these experiments, solutions of oligonucleotides (10 μM), RGG-PLDmut-NLS (10 μM), or their 1:1 mixtures in 40 mM HEPES-KOH buffer (pH 7.0) supplemented with 150 mM KCl were prepared, and ThT was added to a final concentration of 20 μM. ThT fluorescence at 493 nm upon excitation at 450 nm was recorded using a Tecan Infinite 200 Pro plate reader (Swiss Biotech, Basel, Switzerland). All experiments were performed in three to six replicates. ThT was obtained from Sigma-Aldrich (St. Louis, MO, USA).

For free oligonucleotide samples, all ThT signals were normalized to the signal obtained with chap-DNA, which produced the greatest ThT light-up. The resulting normalized values (% of ThT FI_chap-DNA, where FI is fluorescence intensity) are shown as the mean ± SD. The sequence of scr-LNA (CATACGTCTATACGCT), the negative control in ThT assays, was taken from [68].

For the oligonucleotide mixtures with the RGG-PLDmut-NLS peptide, ThT signal normalization was performed as follows:

Norm. ThT signal = (ThT FI_mixture—ThT FI_chaperone)/ThT FI_peptide. The resulting normalized values (% of ThT FI_peptide) are shown as the mean ±SD.

### 4.5. Microscale Thermophoresis (MST)

MST-based assays were used to evaluate the affinity of the oligonucleotides for hnRNP A1. Increasing concentrations of the oligonucleotides were added to a 100 nM solution of the protein labeled with the RED dye. After 10 min of incubation, thermophoretic curves were recorded using fluorescence detection in the RED mode on a Monolith NT.115 instrument (Nanotemper, Munich, Germany). The concentration dependence of the normalized MST signal was approximated by the Hill model in MO Affinity software (version 1) (Nanotemper, Munich, Germany). All experiments were performed in triplicate.

### 4.6. Fluorescence Microscopy

To monitor the formation of biocondensates in solutions containing full-length hnRNP A1 (6 μM), an admixture of its red-labeled derivative (5%), model RNA from Torula yeast (3 mg/mL), and model SR-rich protein SRSF_fr_ (1 mg/mL). These components were dissolved in a 40 mM HEPES-KOH buffer solution (pH 7.0) and supplemented with 150 mM KCl. The crowding agent polyethylene glycol (PEG)-400 [82] was added to induce the formation of dense condensates [38]. The samples were placed in the working chamber between the coverslip and coverslide. After 5–15 min of incubation, the condensates were visualized on an Eclipse Ti2 fluorescence microscope (Nikon, Tokyo, Japan) using fluorescence excitation at 647 nm and a cut-off filter of 660 nm. The volume fraction of condensates in each sample was estimated based on the obtained images using the total area of condensate projections, which was calculated using DropletCalc software (version 1) [71]. All experiments were performed in three replicates.

### 4.7. Atomic Force Microscopy (AFM)

AFM was used to verify the inhibitory effect of the leading oligonucleotide chaperone, chap-LNA, on the amyloidization of the hnRNP A1 fragment, RGG-PLDmut-NLS. For these experiments, solutions of 10 µM RGG-PLDmut-NLS peptide, 10 µM chaperone, and a mixture of the two (at 10 µM each) were prepared in 40 mM HEPES-KOH buffer (pH 7.0), supplemented with 150 mM KCl. The samples were applied to a graphite surface treated with (CH_2_)_n_(NCH_2_CO)_m_-NH_2_ or a mica slice. Scanning was performed according to the previously described protocol [38] using ultra-thin cantilevers [83] and an AFM microscope with a NTEGRA Prima controller (NT-MDT, Moscow, Russia). Filtering and primary analysis of the AFM data were performed using FemtoScan Online software (version 2.3.239 [5.2]) (ATC, Moscow, Russia), and visualization was performed using Gwyddion software (version 2.58). The lengths of the observed objects were calculated using DNA Trace software (version 3.3.6.msi), and all objects longer than 20 nm were designated as fibrils. For quantitative analysis of the fibril fraction, each sample was prepared in at least three replicates on mica, and 100 objects were analyzed in the obtained scans.

## 5. Conclusions

Chaperones that inhibit the aggregation of hnRNP A1 were developed based on DNA/RNA sequences from the human genome and transcriptome recognized by hnRNP A1 in a nonpathogenic, monomeric conformation. These chaperones contain a G4 module as the main functional unit and an RRM-binding motif from ISS as the addressing unit. The modules are connected via a linker duplex from a known aptamer to RGG. Partial backbone modifications were introduced to allow for RNA-like geometry and/or enhanced biostability of the chaperones. According to MST data, chaperones with LNA and 2′-OMe modifications of the addressing module (chap-LNA and chap-OMe) exhibited the highest affinity for hnRNP A1. Their secondary structures were partially confirmed by CD spectroscopy data. Their holdase-like activity was evidenced in studies of hnRNP A1 LLPS and amyloidization using fluorescence microscopy and fluorimetry. Both chap-LNA and chap-OMe suppressed the formation of amyloid-like fibrils and their possible precursors, dense condensates. The interference of chap-LNA with amyloidization was confirmed by AFM data. This chaperon inhibited the assembly of hnRNP A1 fibrils and condensates by over 90%. Thus, it can be considered as a scaffold for further development of hnRNP A1-targeting drug candidates for chaperone therapy of ALS and related aggregation-driven proteinopathies.

## Figures and Tables

**Table 1:** Chaperone sequences and dissociation constants of their complexes with hnRNP A1.

Code	Sequence, 5′→3′ *	Kd_hnRNP A1, μM
chap-RNA	**UCAGUU**GCUGCGGGUGGGUGGGUGGGGCAGC	6 ± 1
chap-DNA	d(**TCAGTT**GCTGCGGGTGGGTGGGTGGGGCAGC)	14 ± 3
cntr-KRAS	d(**TCAGTT**GCTGCAGGGCGGTGTGGGAAGAGGGAAGAGGGGGAGGGCAGC)	15 ± 3
cntr-noISS	d(GCTGCGGGTGGGTGGGTGGGGCAGC)	>100
chap-PS	d(**TCAGTT**GCTGC) ^†^GGGTGGGTGGGTGGG(GCAGC) ^†^	96 ± 7
chap-tPS	d(**TCAGTT**GCTGCGGGTGGGTGGGTGGGGCAGC) ^†^	86 ± 8
chap-LNA	**(TCAGTT**GCTGC) ^‡^GGGTGGGTGGGTGGG(GCAGC) ^‡^	6 ± 5
chap-tLNA	**(TCAGTT**GCTGCGGGTGGGTGGGTGGGGCAGC) ^‡^	14 ± 5
chap-Ome	**(TCAGTT**GCTGC) ^#^GGGTGGGTGGGTGGG(GCAGC) ^#^	6 ± 4
chap-tOMe	**(TCAGTT**GCTGCGGGTGGGTGGGTGGGGCAGC) ^#^	30 ± 10

* Boldface—addressing module; underline—linker; no emphasis—functional module. ^†^ PS; ^‡^ LNA; *^#^* OMe.

## Data Availability

The original contributions presented in the study are included in the article, further inquiries can be directed to the corresponding authors.

## Abbreviations

The following abbreviations are used in this manuscript:

ALS	Amyotrophic lateral sclerosis
hnRNP	Heterogeneous nuclear ribonucleoprotein
RRM	RNA-recognizing motif
EPR	Endoplasmic reticulum
LCR	Low-complexity region
PLD	Prion-like domain
ISS	Intronic splicing silencer
CD	Circular dichroism
MST	Microscale thermophoresis
AFM	Atomic force microscopy
ThT	Thioflavin T
PEG	Polyethylene glycol
